# A comparison of COVID-19, SARS and MERS

**DOI:** 10.7717/peerj.9725

**Published:** 2020-08-19

**Authors:** Tingting Hu, Ying Liu, Mingyi Zhao, Quan Zhuang, Linyong Xu, Qingnan He

**Affiliations:** 1 Department of Pediatrics, The Third Xiangya Hospital, Central South University, Changsha, China; 2 Transplantation Center, The Third Xiangya Hospital, Central South University, Changsha, China; 3 Department of Biomedical Informatics, School of Life Sciences, Central South University, Changsha, China

**Keywords:** COVID-19, SARS-CoV, MERS-CoV, Epidemiology, Clinical presentations, Laboratory diagnosis, Radiological features

## Abstract

In mid-December 2019, a novel atypical pneumonia broke out in Wuhan, Hubei Province, China and was caused by a newly identified coronavirus, initially termed 2019 Novel Coronavirus and subsequently severe acute respiratory syndrome coronavirus 2 (SARS-CoV-2). As of 19 May 2020, a total of 4,731,458 individuals were reported as infected with SARS-CoV-2 among 213 countries, areas or territories with recorded cases, and the overall case-fatality rate was 6.6% (316,169 deaths among 4,731,458 recorded cases), according to the World Health Organization. Studies have shown that SARS-CoV-2 is notably similar to (severe acute respiratory syndrome coronavirus) SARS-CoV that emerged in 2002–2003 and Middle East respiratory syndrome coronavirus (MERS-CoV) that spread during 2012, and these viruses all contributed to global pandemics. The ability of SARS-CoV-2 to rapidly spread a pneumonia-like disease from Hubei Province, China, throughout the world has provoked widespread concern. The main symptoms of coronavirus disease 2019 (COVID-19) include fever, cough, myalgia, fatigue and lower respiratory signs. At present, nucleic acid tests are widely recommended as the optimal method for detecting SARS-CoV-2. However, obstacles remain, including the global shortage of testing kits and the presentation of false negatives. Experts suggest that almost everyone in China is susceptible to SARS-CoV-2 infection, and to date, there are no effective treatments. In light of the references published, this review demonstrates the biological features, spread, diagnosis and treatment of SARS-CoV-2 as a whole and aims to analyse the similarities and differences among SARS-CoV-2, SARS-CoV and MERS-CoV to provide new ideas and suggestions for prevention, diagnosis and clinical treatment.

## Introduction

A large concentration of pneumonia cases in Wuhan city, Hubei Province, China (World Health Organization (WHO), 2020), was first reported by the Wuhan Municipal Health Commission on 31 December 2019 (Paules, Marston & Fauci, 2020). Subsequently, on 30 January 2020, the director-general of the World Health Organization (WHO) proclaimed that the outbreak of the new virus constitutes a Public Health Emergency of International Concern (Liu et al., 2020). The virus, named severe acute respiratory syndrome coronavirus 2 (SARS-CoV-2), can lead to severe pneumonia, and the evidence shows that it can be transmitted from human to human (Huang et al., 2020). By 22 July 2020, SARS-CoV-2 had been detected in approximately 216 countries and regions. Epidemiologists and doctors advised that the infection could be well controlled if people reduce exposure, block transmission and volunteer to isolate themselves while infected. Nevertheless, a number of barriers must be overcome to fight SARS-CoV-2. Accordingly, it is especially necessary to develop stronger preventative measures, improve the public’s awareness of protection, and stop the disease from becoming a pandemic.

Coronaviruses (CoVs) consist of four genera: Alphacoronavirus (α), Betacoronavirus (β), Gammacoronavirus (γ) and Deltacoronavirus (δ). Among these, α and β only infect mammals, whereas γ and δ mainly infect birds. Betacoronaviruses are further divided into four lineages: A, B, C and D. Previously, there have been six human coronaviruses: 229E, HKU1, OC43, NL63, middle east respiratory syndrome coronavirus (MERS-CoV) and severe acute respiratory syndrome coronavirus (SARS-CoV). Among these lineages, 229E, HKU1, OC43 and NL63 are low pathogenic CoVs, whereas SARS-CoV and MERS-CoV are highly pathogenic CoVs (Cui, Li & Shi, 2019). Prior to the SARS-CoV-2 epidemic, there was a devastating outbreak of SARS in 2003 and a similarly devastating outbreak of MERS in 2012, both resulting in up to thousands of deaths; additionally, these outbreaks resulted from zoonotic coronavirus, leading to high morbidity and mortality in the infected population (Liu et al., 2020; Otter et al., 2016).

In this review, we summarize recent research in the field of SARS-CoV-2, especially the biological and epidemiological characteristics, symptoms, laboratory diagnosis, radiological features, treatment and pathology of this virus and present our recommendations. Some of the biological and epidemiological characteristics of SARS-CoV-2 and the clinical presentations that it causes resemble those of SARS-CoV and MERS-CoV; thus, a comparison of the similarities and differences among the three coronaviruses might greatly help minimize the number of deaths caused by SARS-CoV-2 and guide future studies on the new virus.

## Survey methodology

**Figure 1:**
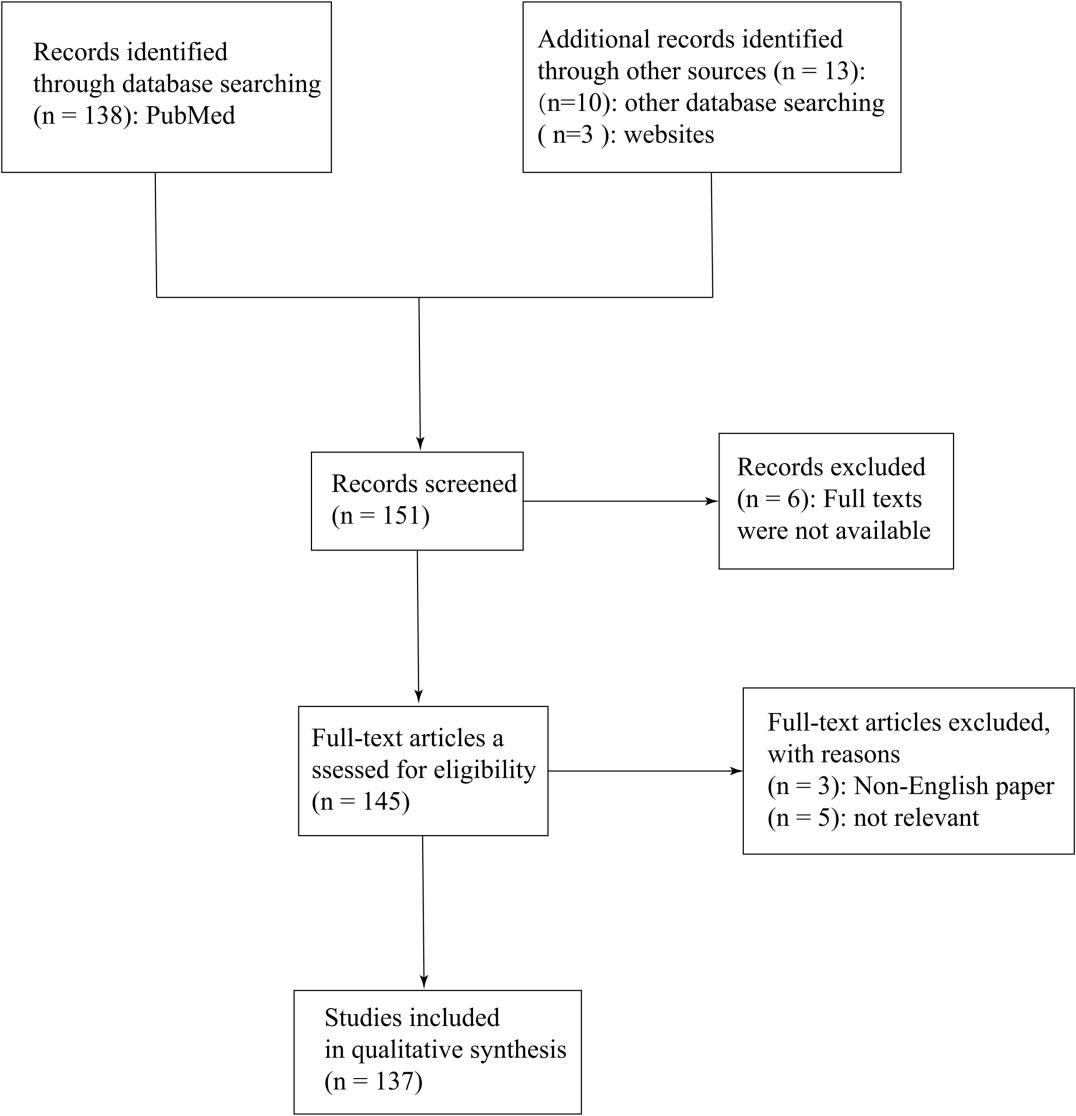
Methodical approach of this review.

First, we set up an independent literature review team to searched for and referred to the guidelines related to SARS-CoV, MERS-CoV and SARS-CoV-2. Wealso consulted the newly released guidelines for SARS-CoV-2 composed by the National Health Commission of People’s Republic of China and the WHO. Then we confirmed the quesition which we would focus on: Are there similarities and differences among COVID-19, SARS and MERS? In addition, recent researcs in the field of SARS-CoV-2 are also in the spotlight by us. Databank and online books were searched on 22 July 2020. During the literature review, we searched the following bibliographic databases: PubMed (mostly), Wiley Online Library and Elsevier/Science Direct. We also searched the following websites: the Centers for Disease Control and Prevention (CDC, https://www.cdc.gov/), the WHO (https://www.who.int/), the National Administration of Traditional Chinese Medicine (http://www.satcm.gov.cn/) and the National Health Commission of the People’s Republic of China (http://www.nhc.gov.cn/). The following search parameters and MeSH terms “SARS-CoV-2”, “SARS-CoV”, “MERS-CoV”, “epidemiology”, “clinical presentations”. “laboratory diagnosis” and “radiological features” delivered 151 hits (see Fig. 1).

We focused on the similarities and differences of COVID-19, SARS and MERS in all respects to be clinically relevant to a great extent. Thus, inclusion criteria were the following:

- Diagnosis of COVID-19, SARS and MERS by medical experts
- Biological studies of SARS-CoV-2, SARS-CoV and MERS-CoV
- Collection and analysis of at least one of the following biomaterials: faecal, urinary or blood samples of confirmed/suspected individuals
- Analysis of isolated SARS-CoV-2, SARS-CoV or/and MERS-CoV by sequencing et al.
- Epidemiological investigations and studies of SARS-CoV-2, SARS-CoV and MERS-CoV
- The latest diagnostic criteria of COVID-19, SARS and MERS including clinical presentations, labora tory diagnosis and radiological feature
- Latest treatment and prevention methods of COVID-19
- Published in a peer-reviewed article
- Availability of the full text publication
- Availability of the paper in English

Most of the included papers were published between 2005 and 2020 and the evalutation of papers was conducted in the procedure above by two experienced researchers. Among the included papers, six papers were excluded because the full texts were not available; besides, three non-English papers were excluded and five papers were also excluded for lacking of any relevance to the subject. Totally, 137 texts were selected for this literature Review.

## Biological and epidemiologic features of the three viruses

### Biological features

Researchers determined the sequence of SARS-CoV-2 through high-throughput sequencing. In addition to the published references on SARS-CoV and MERS-CoV, we compared the biological features of SARS-CoV-2, SARS-CoV and MERS-CoV, which are summarized in Table 1. As shown in this table, SARS-CoV-2 can cause severe acute respiratory syndrome and has a 4.2% mortality rate; similarly, SARS-CoV and MERS-CoV can cause severe acute respiratory syndrome and have mortality rates of 11% and 34%, respectively (Yang et al., 2020).

**Table 1:** A comparison of biological features among SARS-CoV-2, SARS-CoV and MERS-CoV. The data are from the following studies: Zhou et al. (2020), Lu et al. (2020), Chan et al. (2020), Belouzard, Chu & Whittaker (2009), Hui (2013), Wang et al. (2013) and Yang et al. (2020).

Coronavirus			
	SARS-CoV-2	SARS-CoV	MERS-CoV
Homology*	–	79.5%	40%
Possible natural reservoir	Bat (Zhou et al., 2020; Lu et al., 2020)	Bat (Zhou et al., 2020; Lu et al., 2020)	Bat (Zhou et al., 2020; Lu et al., 2020)
Possible intermediate host	Malayan Pangolins and turtles (Zhou et al., 2020; Lu et al., 2020)	Palm civets (Zhou et al., 2020; Lu et al., 2020)	Camel (Zhou et al., 2020; Lu et al., 2020)
The lineage of Betacoronaviruses	B (Zhou et al., 2020; Lu et al., 2020)	B (Zhou et al., 2020; Lu et al., 2020)	C (Zhou et al., 2020; Lu et al., 2020)
Predominant cellulart receptor	ACE2 (Belouzard, Chu & Whittaker, 2009; Hui, 2013)	ACE2 (Belouzard, Chu & Whittaker, 2009; Hui, 2013)	Dipeptidyl peptidase 4 (DPP4, also known as CD26) (Wang et al., 2013)
Symptoms	Severe acute respiratory syndrome, 4.2% mortality rate (Yang et al., 2020)	Severe acute respiratory syndrome, 11% mortality rate (Yang et al., 2020)	Severe acute respiratory syndrome, 34% mortality rate (Yang et al., 2020)

**Note:**

* Homology with SARS-CoV-2.

According to previous studies, SARS-CoV-2 has been confirmed to share 79.5% sequence identity with SARS-CoV and 94.6% sequence identity with SARS-CoV in ORF1a/b; the sequencing results were used for CoV species classification and revealed that both of these viruses are lineage B betacoronaviruses (Zhou et al., 2020; Lu et al., 2020). However, SARS-CoV-2 shares only 40% sequence identity with MERS-CoV, which is a lineage C betacoronavirus (Chan et al., 2020). Civets are the intermediate hosts of SARS-CoV, whereas dromedary camels are the intermediate hosts of MERS-CoV (Zhou et al., 2020; Lu et al., 2020). However, the intermediate hosts of SARS-CoV-2 have not been determined. At present, the prevailing viewpoints suggest Malayan pangolins and turtles (Liu et al., 2020).

**Figure 2:**
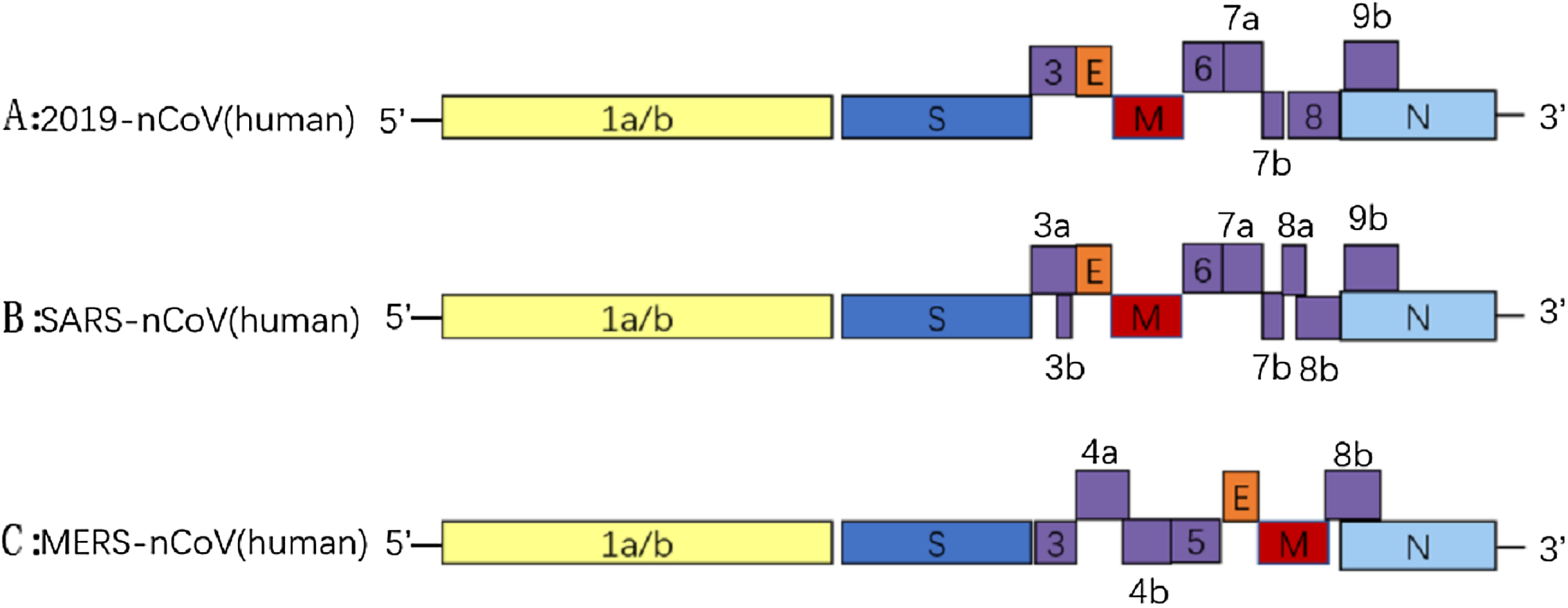
The genome organizations of 2019-nCoV (A), SARS-CoV (B) and MERS-CoV (C).

**Figure 3:**
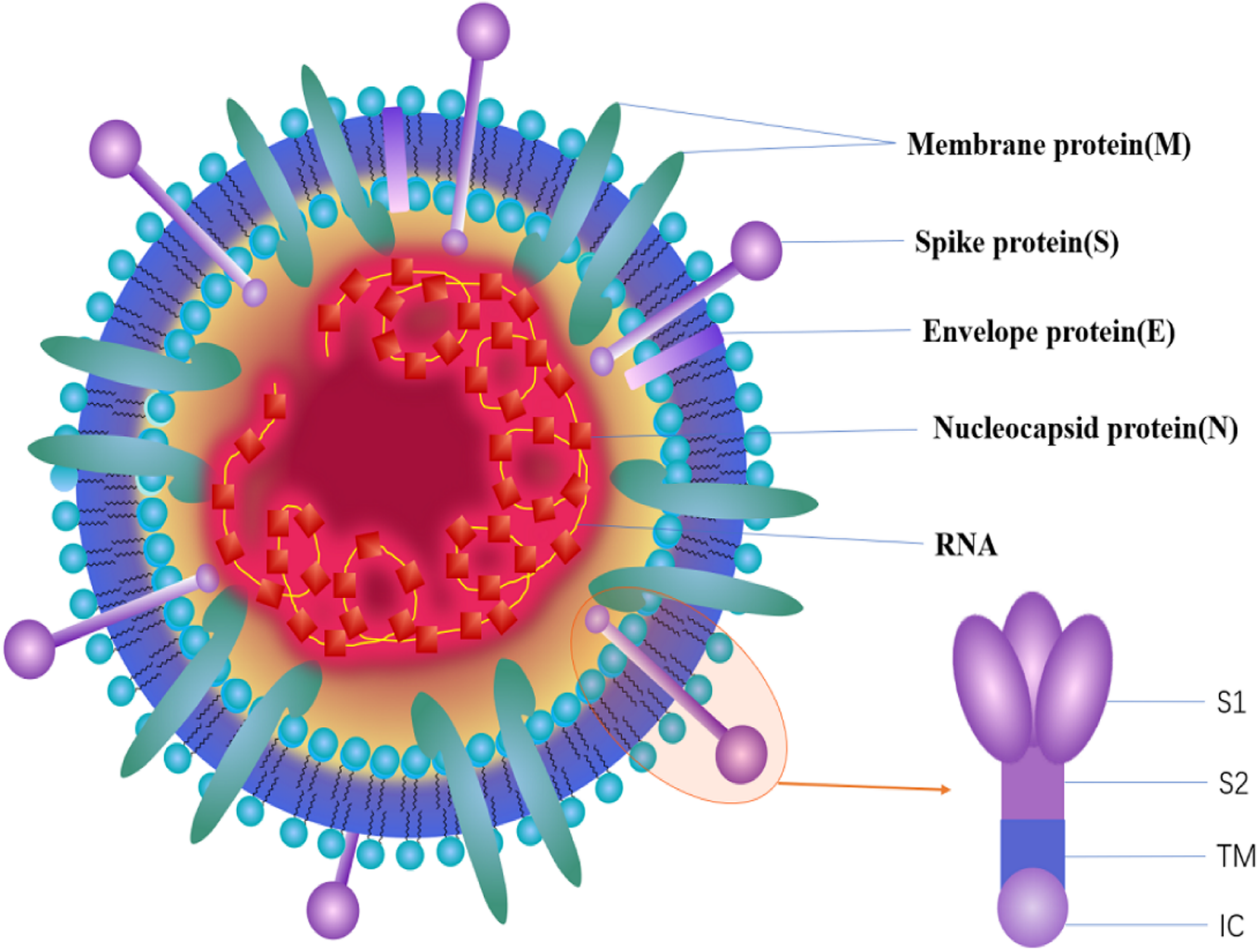
The structure of the coronavirus.

CoVs are RNA viruses characterized by a single-stranded, 5′-capped, positive strand RNA molecule ranging from 26 to 32 kb, and the RNA includes at least six open reading frames (ORFs). The first ORF (ORF1a/b) comprises approximately 2/3 of the genome and encodes replicase proteins, and the remaining ORFs mainly encode four structural proteins: spike (S), membrane (M), envelope (E) and nucleocapsid (N) (Perlman & Netland, 2009). The genome organization of SARS-CoV-2, SARS-CoV and MERS-CoV are described in Fig. 2, and the major distinctions between SARS-CoV-2 and SARS-CoV are in open reading frame-3b (orf3b), spike and open reading frame-8 (orf8), especially in spike S1 and orf8 (Perlman & Netland, 2009). Orf8 is an accessory protein. The SARS-CoV orf8b can activate NLRP3 inflammasomes and trigger pyroptotic cell death, whereas the new orf8 of SARS-CoV-2 does not contain a known functional domain or motif (Shi et al., 2019). The spike protein can mediate coronavirus entry into host cells. This protein has three segments: a short intracellular tail, a single-pass transmembrane anchor (TM) and a large ectodomain (IC), which consists of two subunits (S1 and S2). The S1 subunit can recognize and bind to receptors, and when S1 binds to a host receptor, the S2 subunit is exposed and cleaved by host proteases, mediating viral and host membrane fusion (Li, 2016; Belouzard, Chu & Whittaker, 2009). The coronavirus structure is described in Fig. 3. The N protein is important for the virus capsid and modulates the initial innate immune response by inhibiting type I interferon (IFN) production (Hui, 2013). The M protein and the E protein are involved in virus morphogenesis, assembly and budding. The S protein mediates virus entry into cells. The receptor of SARS-CoV-2 is angiotensin converting enzyme II (ACE2), which is also the receptor of SARS-CoV. ACE2 binds to the SARS-CoV-2 S ectodomain with 15 nM affinity, which is approximately 10- to 20-fold higher affinity than ACE2 binding to SARS-CoV S. In contrast, the receptor of MERS-CoV is DPP4 (Wrapp et al., 2020). Both ACE2 and DPP4 are recognized by the C-terminal domain in S1 (S1-CTD) as a receptor-binding domain (RBD). As previously mentioned, S1 is one of the major distinctions between SARS-CoV-2 and SARS-CoV. These realizations lead one to wonder how these viruses can recognize the same receptor of the host cell despite their inherent differences. The RBD of SARS-CoV contains two subdomains: a core and an extended loop. The core is constructed of a five-stranded anti-parallel β sheet (β1 to β4 and β7) and three short connecting α helices (αA to αC). The extended loop subdomain is positioned to one side of the core, and a two-stranded β sheet (β5 and β6) forms a gently concave outer surface, the base of which cradles the N-terminal helix of ACE2 (Wang et al., 2013). Although only 9 out of 13 glycans in the S1 subunit are conserved among SARS-CoV-2 S and SARS-CoV S, their overall structures are similar, and the most notable difference is the position of the RBDs in their respective down conformations: tightly against the N-terminal domain (NTD) in SARS-CoV and angled closer to the central cavity of the trimer in SARS-CoV-2 (Wang et al., 2013). In contrast to SARS-CoV and other SARSr-CoVs, there is a four amino acid residue insertion at the S1/S2 boundary of SARS-CoV-2 S that results in the presence of a furin cleavage site (Walls et al., 2020). The S1–S2 site cleaved during biosynthesis is not necessary for S-mediated entry, but it may contribute to the high affinity of SARS-CoV-2 S for human ACE2 (Letko, Marzi & Munster, 2020).

Angiotensin converting enzyme II is the receptor of SARS-CoV-2 and SARS-CoV, and it is one of the enzymes in the renin angiotensin system (RAS). It can convert angiotensin I (AngI) to angiotensin 1–9 (Ang 1–9) and angiotensin II (Ang II) to angiotensin 1–7 (ANG 1–7) to decrease AngII, which can increase aldosterone and vasopressin secretion, cause vasoconstriction, and induce myocardial and renal fibrosis (Flores-Muñoz et al., 2011; Serfozo et al., 2020). As counterregulatory components of the ACE-Ang II-AT1 axis, ACE2 and (ANG 1–7) can control inflammation and fibrosis in cardiovascular and renal disease (Simões e Silva & Teixeira, 2016). Expression of the ACE2 receptor is found in many tissues, including lung, heart, kidney, liver, endothelium, intestine, oral mucosa and even testis (Xu et al., 2020; Cheng, Wang & Wang, 2020; Venkatakrishnan et al., 2020; Xu et al., 2020). ACE2 is reported to improve acute lung injury, suppress hypertension and cardiac dysfunction, reduce glomerular and biliary fibrosis, stimulate brown adipose tissue and induce browning in white adipose tissue (Minato et al., 2020; Liu et al., 2020; Rajapaksha et al., 2019; Kawabe et al., 2019; Chen et al., 2019). All these factors could be targets for SARS-CoV-2 to damage human health. In addition, the level of ACE2 was reported to be related to IL-6, which plays an important role in cytokine storm syndrome (CSS), by Long Chen1 and Li Zhong on Preprint.org. CSS is considered to increase the illness severity of SARS-CoV-2 (Huang et al., 2020).

The lungs are the main target organ of SARS-CoV-2. According to recent research, ACE2 is expressed in 0.64% of all human lung cells, and the majority of them are type II alveolar cells (AT2) (average 83%) (Huang et al., 2020). Consistent with these findings, SARS-CoV-2 presents as lesions involving mainly destruction of the distal alveoli. Other lung cells, such as type I alveolar cells (AT1), endothelial cells, airway epithelial cells, fibroblasts and macrophages, were also reported to express ACE2. Though their ratio is low and variable among individuals, they may also be the target of SARS-CoV-2 (Hamming et al., 2004).

Severe acute respiratory syndrome coronavirus 2 also causes gastrointestinal symptoms, and approximately 3% of patients develop diarrhoeal symptoms (Holshue et al., 2020). Accordingly, oesophageal upper and stratified epithelial cells as well as absorptive enterocytes from the ileum and colon were also reported to be ACE2 positive (Yang et al., 2020). SARS-CoV-2 was also reported by the Third People’s Hospital of Shenzhen to be present in faecal samples. Based on the above-mentioned studies, many researchers speculated thatthe faecal-oral route may be one route of SARS-CoV-2 transmission; however, it has been fairly settled by WHO that the fecal-oral route does not drive transmission and we should not read too much into this mode of transmission. According to several recent studies, acute kidney injury (AKI) has been reported in over 20% of patients who suffered from COVID-19 in China and the US (Yang et al., 2020; Richardson et al., 2020). Besides, a meta-analysis showed the prevalence of chronic kidney disease was higher in severe patients with COVID-19 (3.3% vs. 0.4%; odds ratio 3.03, 95% CI [1.09–8.47]) (Henry & Lippi, 2020). This outcome may be attributed to ACE2 because ACE2 is expressed in the kidney, especially in proximal tubular cells and bile duct cells (Wu et al., 2018; MacParland et al., 2018; Soler et al., 2019). Notely, ACE2 is also expressed by endothelial cells, and other major clinical events commonly observed in COVID-19 patients including high blood pressure, thrombosis, pulmonary embolism, cerebrovascular and neurologic disorders (Lovren et al., 2008; Chen et al., 2020; Poissy et al., 2020; Aggarwal, Lippi & Michael Henry, 2020; Mao et al., 2020; Baumgartner-Parzer & Waldhausl, 2001).

### Epidemiologic features of the three viruses

The SARS-CoV-2 pneumonia outbreak in Wuhan began in early December 2019. Among the first 41 laboratory-confirmed patients, 27 (66%) had been exposed to the Huanan seafood market, where wild animals are sold, suggesting that COVID-19 may be passed to humans from wild animals (Huang et al., 2020). The median incubation period of COVID-19 is 3.0 days (range, 0–24.0 days) (Chen et al., 2020). The disease is highly contagious, and the speed of the spread and the infectivity of COVID-19 dramatically exceeded those of MERS-CoV and SARS-CoV (Goh et al., 2020). The basic reproduction number (R0) reflects the rate of disease transmission. Recent data revealed an R0 for COVID-19 of 2.56 (95% CI [2.49–2.63]), indicating that one patient could transmit the disease to 2.56 other people (Zhao et al., 2020). According to recent data from the WHO, Centers for Disease Control and Prevention (CDC), and reports to the WHO from various countries and their allied agencies, as of 22 July 2020, a total of 14,562,550 individuals were reported as infected with SARS-CoV-2 among 216 countries, areas or territories with recorded cases, and the overall case-fatality rate (CFR) was 4.2% (607,781 deaths among 14,562,550 recorded cases). In a report from the WHO (https://www.who.int/emergencies/diseases/novel-coronavirus-2019/situation-reports), Europe and the Americas became the locations of the most serious epidemics, instead of China, which had previously been the location for the most serious outbreaks. In China, people with overseas exposures have become the high-risk population, as with Wuhan-related exposures. Male sex and older age are two significant risk factors (Shen et al., 2020). According to a study among 138 hospitalized patients with 2019 novel coronavirus-infected pneumonia in Wuhan, China, the male-to-female ratio was 1.06:1, and the median age was 56 years (interquartile range, 42–68; range, 22–92 years) (Wu & McGoogan, 2020; Wang et al., 2020). Of the 1,591 patients requiring treatment in an intensive care unit (ICU) in the Lombardy region of Italy, the median (IQR) age was 63 (Richardson et al., 2020; Borghesi et al., 2020; Zheng et al., 2020; De Wit et al., 2016; Gao et al., 2016) years, and 1,304 (82%) were male (Grasselli et al., 2020). According to the data from 5,700 hospitalized patients with confirmed COVID-19 in New York City, the median age was 63 years (interquartile range, 52–75; range, 0–107 years), and 39.7% were female (Richardson et al., 2020). One study showed that males aged 50 years or older and females aged 80 years or older showed the highest risk (Borghesi et al., 2020). Health workers are one of the high-risk groups. A total of 3.8% of confirmed COVID-19 patients were health workers (Aggarwal, Lippi & Michael Henry, 2020). Diabetes could be one of the risk factors for progression to severe/critical outcomes (Zheng et al., 2020). Human–human transmission is considered a major transmission mode of COVID-19 by the sixth version of the Guidance for Diagnosis and Treatments for COVID-19 issued by the National Health Commission of China. And according to the latest reports from the WHO, the driving transmission of COVID-19 contains droplet transmission, contact transmission and aerosol transmission.

From November 2002, when the first known case of SARS occurred in Foshan, China, to July 2003, when the WHO declared the SARS pandemic over, a total of 8,096 cases were reported in 27 countries, including 774 deaths for a CFR of 9.6% (De Wit et al., 2016). Among these 8,096 cases, 23.1% were health workers, the male–female ratio was 1:1.25, and the mean age was 39.9 years, ranging from 1 to 91 (Rota et al., 2003). The mean incubation period was 6.4 days (range 2–10) (Park et al., 2019). Carrying an HLACw*0801 allele is a risk factor for SARS (Chen et al., 2006). Older age and male gender were predictive of poor prognosis (Chan, Tsui & Wong, 2007). Human–human transmission is also the major transmission mode of SARS-CoV, and the primary route of transmission for SARS is contact of the mucous membranes with respiratory droplets or fomites (https://www.who.int/zh).

MERS first emerged in September 2012 in Saudi Arabia and is still not contained. According to data from the WHO (https://www.who.int/csr/don/24-february-2020-mers-saudi-arabia/zh/), as of 31 January 2020, 2,519 cases of MERS had been laboratory-confirmed, including 858 associated deaths (CFR: 34.4%) that were reported in the 27 countries globally that have had cases of MERS-CoV; the proportion of healthcare workers infected in Saudi Arabia from January 2013 to November 2019 was 19.1%. Based on the analysis of WHO data from 23 September 2012 to 18 June 2018, Ahmadzadeh et al. (2020) described the epidemiological characteristic of MERS as follows: the CFR was 32% (95% CI [29.4–34.5]); the male-to-female ratio was 2.52; the mean age was 50.21 ± 18.73 years and ranged from 2 to 109 years of age; and the risk factors for MERS included age >30 years old, Saudi nationality, comorbidities, and the interval time from symptom onset and admission to the hospital >14 days. The median incubation period was seven days (range 2–17) (Cho et al., 2016). Human–human transmission in MERS-CoV is limited and not sustained (Bernard-Stoecklin et al., 2019). Camels were thought to be involved in its spread to humans, but in what way remains unclear. According to a report from the WHO (https://www.who.int/news-room/fact-sheets/detail/middle-east-respiratory-syndrome-coronavirus-(mers-cov)), people become infected through unprotected contact with infected dromedary camels or infected people. In conclusion, we summarized the epidemiological comparisons of COVID-19 with SARS and MERS in Table 2.

**Table 2:** Epidemiological comparison of COVID-19 with SARS and MERS. The data are from the following studies: Poissy et al. (2020), Aggarwal, Lippi & Michael Henry (2020), Mao et al. (2020), Baumgartner-Parzer & Waldhausl (2001), Huang et al. (2020), Chen et al. (2020), Goh et al. (2020), Zhao et al. (2020), Shen et al. (2020), Wu & McGoogan (2020), Wang et al. (2020), Grasselli et al. (2020), Richardson et al. (2020) and Borghesi et al. (2020). Other data were obtained the World Health Organization (WHO, https://www.who.int/emergencies/diseases/novel-coronavirus-2019), the Centers for Disease Control and Prevention (CDC, https://www.cdc.gov/coronavirus/2019-ncov/cases-updates/index.html), and reports from various countries and their allied ministries to the WHO (https://www.who.int/emergencies/diseases/novel-coronavirus-2019/events-as-they-happen; https://www.worldometers.info/coronavirus/?utm_campaign=homeAdvegas1; https://www.ecdc.europa.eu/en/covid-19-pandemic).

	2019-nCoV	SARS-CoV	MERS-CoV
Outbreak date	December 2019	November 2002	September 2012
Confirmed cases	4,731,458 (19 May 2020)	8,096 (31 July 2003)	2,519 (31 January 2020)
Total death	316,169 (19 May 2020)	774 (31 July 2003)	866 (31 January 2020)
CRF	6.6%	9.6%	34.4%
Countries, areas or territories with cases	213 (19 May 2020)	29 (31 July 2003)	27 (31 January 2020)
Incubation period, days (range)	3.0 (0–24.0)	6.4 (2–10)	7 (2–17)
Major routes of transmission	Respiratory aspirates, droplets, contacts and feces	Respiratory aspirates, droplets and contacts (World Health, 2003)	Unprotected contact with infected dromedary camels or infected people
Age, years (range)	56 (22–92) (in Wuhan, China)	39.9 (1–91)	50.21 (2–109)
	63 (56–70) (in an ICU in the Lombardy region of Italy)		
	63 (0–107) (in the New York City)		
Proportion of health workers	3.8%	23.1%	19.1%
Male:female ratio	1.06:1	1: 1.25	1:2.52 (Ahmadzadeh et al., 2020)
Risk areas	Europe, Americas	China	Saudi Arabia
Risk factors	Male, older ags	Cw*0801 HLA	Age >30 years old, Saudi nationality, comorbidities, the interval time of onset sign and the admission to the hospital >14 days

## Symptoms of covid-19, sars and mers

COVID-19, SARS, and MERS show several similarities regarding their symptoms, which include fever, cough, myalgia, fatigue and lower respiratory signs. However, the symptoms vary with the state of the illness and in the process of disease progression. Notably, 60% of patients suffering from SARS have watery diarrhoea in addition to the abovementioned symptoms with the characteristics that there is a representative biphasic clinical course (Hui & Zumla, 2019; Monaghan, 2016). Patients who suffer from MERS have symptoms that include fever, cough (predominantly dry), malaise, myalgia, nausea, vomiting, diarrhoea, headache and even renal failure (Arabi et al., 2014; Gao et al., 2016). Not surprisingly, the symptoms of MERS resemble SARS, but the clinical course is unpredictable and changeable. More than half of the MERS patients are reported to develop acute renal damage at an average time of approximately ten days after the onset of symptoms; additionally, the majority of the cases require renal replacement therapy (Arabi et al., 2014; Assiri et al., 2013; Memish et al., 2014). However, approximately 6.6% of patients who are infected with SARS develop acute renal failure at a median time of 20 days after onset, and only 5% call for replacement therapy (Chan, Tsui & Wong, 2007). Direct renal injury is considered to contribute to the common symptoms of acute renaldamage in MERS patients (Monaghan, 2016); namely, DPP4 is present in renal cells, and MERS-CoV can be detected in urine. The majority of patients with COVID-19 infections present with fever (98%), cough (76%), and myalgia or fatigue (44%). It has been reported that 55% of patients can present with dyspnoea, which develops a median of eight days after the onset of initial symptoms (Kanne, 2020). In light of the studies from all over the country, the symptoms suggest that the target cell is likely present in the lower respiratory tract, as patients who are infected with COVID-19 seldom have conspicuous upper respiratory symptoms such as sneezing or sore throat (Huang et al., 2020). The autopsy reports of new coronavirus pneumonia (NCP) patients indicate that the disease mainly causes distal airway inflammatory reactions and alveolar damage, which is coincidental with the abovementioned symptoms. The fibrosis and consolidation in the lungs of SARS patients are more serious than the lesions caused by COVID-19, which indicates that the chest lesions are not primarily serous inflammation; instead, the exudative reaction of SARS is less than that of COVID-19.

## Laboratory diagnosis and radiological features

### Laboratory diagnosis

#### Specimens

Laboratory diagnosis plays a leading role in the early detection of infected individuals, which enables an earlier discovery of the source of infection and interruption of epidemic transmission (Yan, Chang & Wang, 2020). The first and crucial step for laboratory testing is the collection and processing of suitable specimens. In light of the published studies, viral RNA, considered one of the gold standards of detection, can be found in the upper respiratory tract (URT), lower respiratory tract (LRT), stool, blood, and urine of patients who are infected with SARS-CoV, MERS-CoV, and SARS-CoV-2. The detection rates of the abovementioned specimens for SARS-CoV, MERS-CoV, and SARS-CoV-2 infection are summarized in Table 3 (Zhang et al., 2020; Young et al., 2020; Peiris et al., 2003; Poon et al., 2003; Corman et al., 2016; Ng et al., 2003; Chen et al., 2020; Ling et al., 2020). We can select the specimens showing higher sensitivity in different stages.

**Table 3:** The detection rates of SARS-CoV-2, MERS-CoV, and SARS- CoV infection. Data are converted directly from raw data or estimated from figures. Data are all from the following studies: Hui & Zumla (2019), Monaghan (2016), Arabi et al. (2014), Gao et al. (2016), Assiri et al. (2013), Memish et al. (2014), Kanne (2020) and Huang et al. (2020).

Sample type	Time of illness (days)	SARS-CoV-2		MERS-CoV (Memish et al., 2014; Huang et al., 2020)		SARS-CoV	
URT	7	Throat swabs	Nasopharyngeal swabs	Throat swabs	Nasopharyngeal swabs	NP aspirates	Other URT specimens ①
		50% (Arabi et al., 2014)	100% (Gao et al., 2016)	75.5%	33.3%	32%^a^ (Arabi et al., 2014)	39% (Memish et al., 2014)
						80%^b^ (Gao et al., 2016)	
	14	25% (Arabi et al., 2014)	94.4% (Gao et al., 2016)	45.8%	23.5%	68% (Arabi et al., 2014)	32% (Hui & Zumla, 2019)
	21	N/A	69.2% (Gao et al., 2016)	0	5.88%	39% (Arabi et al., 2014)	25% (Hui & Zumla, 2019)
LRT (sputum or tracheal aspirate)	7	N/A		100%		100% (Hui & Zumla, 2019)	
	14	N/A		93%		100% (Hui & Zumla, 2019)	
	21	N/A		66.7%		67% (Hui & Zumla, 2019)	
Blood	7	40% (Arabi et al., 2014)		10%		50% (Assiri et al., 2013)	
	14	25% (73)		29.4%		50% (72)	
	21	N/A		17.6%		25% (Assiri et al., 2013)	
Urine	7	6.9% (74)		9%		33% (67)	
	14	N/A		0		25% (Hui & Zumla, 2019)	
	21	N/A		0		14% (Hui & Zumla, 2019)	
Stool	7	53% (Monaghan, 2016)		16.7%		47% (Hui & Zumla, 2019)	
	14	37.5% (69)		14.3%		97% (Arabi et al., 2014)	
	21	N/A		0		54% (Hui & Zumla, 2019)	

**Notes:**

a Conventional RT-PCR.

b RT-PCR assay.

N/A, not applicable, that is, the lack of data in a form or table.

Other URT specimens: Throat swabs and nasopharyngeal swabs.

#### Nucleic acid tests

Currently, nucleic acid tests (NAT) are widely considered the optimal method for diagnosis, since specific primers and standard operation procedures have been established during sequencing of the total genome of the coronavirus (Monaghan, 2016). In general, real-time polymerase chain reaction (RT-PCR) is thought to be the preferred and most widely used NAT method (Gootenberg et al., 2017). The ORF1a, ORF1b, S gene, and N gene, in addition to the M gene and 3′UTR, are all gene targets of RT-PCR assays, which can have high sensitivity. The RT-PCR methods for SARS-CoV, MERS-CoV and SARS-CoV-2 varied in genome target, sequence, assay use, etc. (Lu et al., 2014; Emery et al., 2004; Yan, Chang & Wang, 2020). As shown, most of the in-house assays, as well as commercial kits, can detect two or three regions of the virus genome. However, there are many knowledge gaps and limitations to overcome, including the difficulties of obtaining testing kits due to the global shortage, the requirements of having access to sophisticated equipment, and the management of false negatives that need to be retested (Al-Tawfiq & Memish, 2020; Loeffelholz & Tang, 2020).

The abovementioned limitations have resulted in the development in recent years of many kinds of NAT testing kits used for routine detection of SARS-CoV and MERS-CoV, such as nucleic acid sequence-based amplification (NASBA), loop-mediated isothermal amplification (LAMP), rolling-circle amplification (RCA), clustered regularly interspaced short palindromic repeats (CRISPR) system, etc., the details of which are summarized in Table 4. As shown, a real-time NASBA assay was developed by Chantratita et al. (2004) that can detect only one strand of SARS-CoV RNA. In the same year, the RT-LAMP assay for SARS-CoV can detect 0.01 PFU, which is 100-fold greater than RT-PCR sensitivity (Hong et al., 2004). Several RT-LAMP methods of MERS-CoV that can detect 3.4–15.8 copies of MERS-CoV RNA have been developed (Shirato et al., 2018; Lee et al., 2017). RT-PCR can detect SARS-CoV and MERS-CoV with sensitivities of five copies and 10 copies, respectively (Wang et al., 2005; Abd El Wahed et al., 2013).

**Table 4:** New NAT testing kits and the Sensitivity of SARSS-CoV, MERS-CoV and SARS-CoV-2. Data are expressed as “viral load” (log10 copies/mL or PFU). The data are from the following studies: Emery et al. (2004), Yan, Chang & Wang (2020), Al-Tawfiq & Memish (2020) and Loeffelholz & Tang (2020).

	SARS-CoV	MERS-CoV
NASRA	One copy	N/A
RT-LAMP	0.1 PFU	3.4–17.8 copies
RT-RCA	Five copies	10 copies

**Note:**

N/A, not applicable, that is, the lack of data in a form or table.

At present, many NAT kits have been developed for SARS-CoV-2, especially RT-PCR. However, according to previous studies, the currently available RT-PCR kits are variable and offer sensitivities ranging between 45 and 60% (Yi, 1990; Al-Tawfiq & Memish, 2020). In addition, these methods are not satisfactory, especially in resource-limited regions. There is no doubt that NAT based on clinical manifestations and epidemiology can be an early and rapid screening diagnosis. If NAT is not available, CT imaging also plays a vital role in the diagnosis.

### Radiological features

Medical imaging technology that is commonly used to diagnose SARS, MERS and COVID-19 includes chest X-ray (CXR), computed tomography (CT) and high resolution computed tomography (HRCT). The spatial resolution of CXR is high, but the density resolution is not very good, which leads to a relatively high omission diagnostic rate. Instead, CT has a higher density resolution because it can quantitatively evaluate images.Not surprisingly, HRCT has a high spatial and density resolution and can detect small parenchymal lesions early.

#### The radiological features of SARS

##### CXR findings

The earliest radiographic abnormalities were described as GGOs, and in the majority of patients, the radiographic abnormalities progressed rapidly to focal, multifocal or diffuse consolidation. Airspace shadowing is the most common abnormality found on the CXR at presentation but it is nonspecific because, according to the survey, 10–40% of symptomatic patients in published reports, irrespective of age, have normal initial CXRs, and the findings may be indistinguishable from pneumonia of other causes (Ooi & Daqing, 2003). In approximately two-thirds of patients, unilateral lung involvement is found at presentation, and as the condition progresses, it can become bilateral with maximal lung involvement. In addition, consolidation tends to be peripheral in distribution with lower zone predominance (Ooi & Daqing, 2003). Moreover, CXR obtained in most SARS patients at initial presentation is abnormal, and the disease progression in these patients can be monitored with serial chest radiography (Paul et al., 2004).

##### CT findings

Ground-glass opacification, consolidation, and inter-lobular interstitial and intralobular septal thickening are the most common findings in HRCT scans, mainly with predominant involvement of the periphery and lower lobe. According to a previous study, high-resolution CT scans (HR-CT) may be useful in detecting opacities in patients with normal CXR (Paul et al., 2004; Groneberg et al., 2003). In early stages, abnormalities described on CT include ground-glass opacification, consolidation, interlobular interstitial and intralobular septal thickening (Wang et al., 2003). When in the progressive stage, the disease findings by HRCT, including lesion size, range and severity, progressed steadily. When in the late stages, consolidation can be indistinguishable from acute respiratory distress syndrome (ARDS). Above all, CT is more sensitive in depicting SARS than conventional CXR, and CT images obtained in patients with normal chest radiographs may show extensive disease and airspace consolidation (Hong et al., 2004).

#### The radiological features of MERS

##### CXR findings

A study by Das et al. (2016) indicated that abnormalities can be detected on initial CXR in 83% of patients who suffered from MERS-CoV, and the main CXR findings of MERS-CoV include ground-glass opacity (66%) and consolidation (18%), both of which are distinct in 16% of infection cases. The consolidation can be patchy, coalesced or show opaque rounded nodules. The primary CXR of infected cases appears as lung parenchymal abnormalities with the dominating images of a peripheral mid-lung zone and peripheral lower lung zone. Involvement of multifocal lung parenchymal abnormalities is less common than unifocal involvement. Furthermore, serial CXR obtained during the progression of the disease can be used to evaluate the degree of radiographic deterioration. In general, the progression of MERS can be divided into four types (i.e., types 1–4). Type 1 is the disease development period that appears as initial radiographic deterioration with improvement. Type 2 is the quiet period that shows no apparent changes in CXR. Type 3 manifests as fluctuating radiographic changes with at least two radiographic peaks divided by a stage of soft remission. Type 4 is the progression of radiographic deterioration (Das et al., 2016).

##### CT findings

On the basis of the statistics and the published references, MERS CT imaging findings are more sensitive than CXR; unquestionably, CT is used to confirm or assess the progress of highly suspected MERS (Das et al., 2016). The most common CT findings of MERS are that of bilateral, predominantly subpleural and basilar airspace changes, with more extensive GGOs than consolidation (Ajlan, 2015). CT scans were also assessed for the presence of consolidation, cavitation, centrilobular nodules, tree-in-bud pattern, septal thickening, perilobular opacities, reticulation, architectural distortion, subpleural bands, traction bronchiectasis, bronchial wall thickening, intrathoracic lymph node enlargement, interlobular thickening and pleural effusions (Das et al., 2015). Generally speaking, ground-glass opacities, consolidation and a combination of the abovementioned image results are commonly found during the first week of infection; additionally, pleural effusion and interlobular thickening can also be seen on CT imaging findings of some infected cases (Das et al., 2015). Other CT imaging findings can be seen in different stages with the development of the disease. Interestingly, Petruzzi, Shanthly & Thakur (2009) proposed that PET/CT, a new imaging technology, can be more sensitive and specific than traditional imaging technology such as CT and CXR. Their research also confirmed that PET/CT can quantitatively describe pulmonary infections. In 2016, a study by Das et al. (2016) proposed that distinctly increased β-2-(18F)-Fluoro-2-deoxy-D-glucose (18F-FDG) might be seen in patients who are infected with MERS (Wang et al., 2005).

#### The radiological features of COVID-19

##### CXR findings

In the early phase, CXR of COVID-19 patients is not highly recommended for clinical diagnosis because of its low sensitivity in detecting SARS-CoV-2 pneumonia. Nevertheless, in some areas with limited resources, CXR, as a radiological technology, might have some practical applicability (Paul et al., 2004). The earliest radiographic abnormalities can be negative or only a few patchy increased density shadows. As the disease progresses, CXR can detect double lung patterns and patchy increased density shadows, which are mainly in the lower bilateral lung fields. Furthermore, severely ill patients can exhibit diffuse consolidation of both lungs, even showing a “white lung” (Yang et al., 2020; Pan et al., 2020; Jakobsson et al., 2014).

##### CT findings

The imaging findings of COVID-19 are different regarding the age of the patients, the stages of disease, etc. (Li & Xia, 2020). The CT imaging findings of COVID-19 are divided into early stage, progressive stage and severe stage considering the scope and evolution of lesions, and the characteristics of each stage are as follows: (1) Early stage: single or multiple scattered patchy or conglomerate GGO, mainly in the middle and lower lungs and along the bronchovascular bundles; (2) Progressive stage: increase of imaging fingdings in density; Extent coexisting with the new areas of disease; Consolidation growing with air bronchograms.; (3) Severe stage: diffuse consolidation of the lungs of varying density; The fibrous exudating; Air-bronchograms; Bronchial dilation.; (4) Dissipation stage: gradual resolution of the GGO; Consolidation in the lungs with some residual curvilinear opacities compatible with fibrosis (Kanne, 2020; Wu et al., 2020; Jin et al., 2020). The CT imaging features of lesions are as follows: (1) The predominant distribution is in the subpleural region along the bronchial tract; (2) Single and double lesions are rarely seen on CT scans, instead, multiple lesions are more common; (3) The lesions can be patchy, nodular, lumpy, honeycomb-like, etc.; (4) The density is commonly uneven, and GGOs, interlobular thickening, consolidation, crazy-paving change, etc. can be seen on CT scans; and (5) Pleural effusion is rarely seen, but concomitant signs, such as air bronchogram, in addition to mediastinal lymph node enlargement, are common. Many published studies have indicated that chest CT should be considered as a major role in the diagnosis and management of COVID-19. For instance, research by Li. Y found that chest CT has a low false negative rate in detecting COVID-19 (3.9% 2/51 cases); thus, a negative chest CT might aid in the management of COVID-19, that is, guide the doctors to make a decision whether the patient should be isolated during the incubation window (Das et al., 2016). However, some researchers still raise objections that a negative chest CT does not ensure that a person is not infected with SARS-CoV-2; equally, the abnormalities of the chest CT are not exclusive for COVID-19 (Adair & Ledermann, 2020). In general, we should use chest CT reasonably, and there is doubt that early diagnosis based on chest CT can contribute to overcoming the outbreak as soon as possible.

#### A summary and a comparison of the three kinds of coronavirus pneumonia

COVID-19 pneumonia tends to show similarities with SARS and MERS pneumonia on CXR, with a predominance of bilateral ground-glass opacities and consolidative lesions in the peripheral lung. COVID-19 seems radiologically milder than SARS and MERS despite the similarities in CT findings (Yoon et al., 2020). Each disease undoubtedly has unique radiological features, and the summary data are in Table 5 (Kanne, 2020; Das et al., 2015; Wang et al., 2003). In addition, changes in radiographic scores of SARS in general tended to reflect temporal changes in clinical and laboratory parameters such as oxygen saturation (SaO2). Moreover, there were significant inverse relationships between radiographic scores and SaO2. In a study involving a cohort of patients with serologically confirmed SARS, all patients with diffuse consolidation at maximal radiographic change required oxygen supplementation, including mechanical ventilation (Yang et al., 2020). Therefore, we can speculate that it may also be suitable for COVID-19.

**Table 5:** CT findings of COVID-19 SARS and MERS. Data are *n* (%) unless otherwise stated. The date of COVID-19 is from the review by Kanne (2020). The date of MERS is from the review by Das et al. (2016). The date of SARS is from the article by Wang et al. (2003).

CT findings	COVID-19	SARS	MERS
Ground-glass opacity	86%	81.8%	86.6%
Consolidation	29%	45.5%	33.3%
Crazy-paving	19%	36.4%	27%
Linear	14%	0	0
Bronchiectasis	0	18.2%	0
Interlobular thickening	0	N/A	40%
Pleural effusion	0	22.7%	60%
Pulmonary fibrosis with emphysema	0	0	6.6%
Position on CT			
Peripheral distribution	33%	69%	54%
Central distribution	76%	31%	24%
Mixed	0	0	22%

**Note:**

N/A, not applicable, that is, the lack of data in a form or table.

## Overlapping and discrete aspects of the pathology of sars, mers and covid-19

The available pathology data for SARS and MERS infections mainly rely on limited numbers of autopsy and biopsy cases. According to previous studies, oedematous lungs with increased gross weights and multiple areas of congestion are the predominant visceral macroscopic changes in fatal SARS cases. Moreover, enlargement of the lymph nodes in the pulmonary hila and the abdominal cavity, as well as diminished spleen size and reduced spleen weights, are also the most common changes (Nicholls et al., 2003). Morphological changes of SARS include bronchial epithelial denudation, loss of cilia, and squamous metaplasia (Nicholls et al., 2003; Ding et al., 2003; Liu et al., 2020). In the early phase, the histological features of pulmonary SARS infections may be commonly connected with acute diffuse alveolar damage; on the contrary, a combination of diffuse alveolar damage and acute fibrinous and organizing pneumonia are demonstrated in the later phases of the disease (Gu et al., 2005). Some studies suggest that the pathological features of MERS infection are varied and include exudative diffuse alveolar damage with hyaline membranes, pulmonary oedema, type II pneumocyte hyperplasia, interstitial pneumonia (which is predominantly lymphocytic), and multinucleate syncytial cells. Moreover, bronchial submucosal gland necrosis was also observed, and it includes the pathologic basis for respiratory failure and radiologic abnormalities of MERS infection (Alsaad et al., 2018). Ultra-structurally, viral particles could be found in the pneumocytes, pulmonary macrophages, macrophages infiltrating the skeletal muscles, and renal proximal tubular epithelial cells (Walker, 2016). On 17 February 2020, the team of academician Wang of PLA General Hospital performed a pathologic dissection on a patient who died of COVID-19. Tissue samples were taken from the patient’s lung, liver and heart tissues, and histological examination of the lung revealed bilateral diffuse alveolar injury with fibrous mucinous exudation. In the right lung tissue, prominent alveolar epithelial exfoliation and clear membrane formation suggested ARDS, and in the left lung, tissue pulmonary oedema and clear membrane formation suggested the early stage of ARDS. Mononuclear inflammatory infiltrations of lymphocytes in the stroma were manifested in both lungs, multinucleated giant cells and uncharacteristically enlarged alveolar cells were found in the alveolar exudate, and the latter contained larger nuclei, bi-tropic intracytoplasmic granules and prominent nucleoli, which showed viral cytopathic-like changes. Additionally, no obvious intracytoplasmic or nuclear virus inclusion bodies were found. The severity of pulmonary lesions in SARS, MERS and COVID-19 is different, especially the destruction of alveoli and the degree of necrotic lung. According to the comparison of the pathology, COVID-19 seems to be not as severe as SARS. Indeed, they show some similarities in pathology in that they all have type II pneumocyte hyperplasia. However, they also have some differentia. In general, the pathological features of SARS are that a large proportion of type II pneumocytes fall off into the alveolar cavity, while the pathological features of SARS are that there are many cells proliferating and active in the alveolar walls, and some cells even fall into the lumen in clumps, especially in the late stage (Qu et al., 2020; Xu et al., 2020; Barrett et al., 2020). Indeed, the degree and progression of alveolar fibrosis are varied. In summary, these results suggest that pathology may also play a relevant role in the development of disease severity.

## Treatments of sars, mers and covid-19

During the SARS-CoV epidemic, there were no treatments available to reduce SARS-related diseases and deaths. The earliest treated patients received intravenous injection (IV) ribavirin, which was based on its broad-spectrum antiviral activity, because there was insufficient time to perform efficacy studies (Poutanen et al., 2003). After confirming that SARS-CoV was the causative agent, many studies on treatments for SARS were started. The most commonly used treatments for SARS include ribavirin, LPV/r, corticosteroids, interferon (IFN) and convalescent plasma or immunoglobulins. Unfortunately, the abovementioned treatments are associated with adverse effects, including avascular necrosis and osteoporosis (Stockman et al., 2006). Therapeutically, MERS therapies resemble SARS therapies, among which antiviral therapy has been widely studied. Broad spectrum antiviral drugs with low toxicity, including interferon, ribavirin and cyclophilin inhibitors, which were used effectively for SARS, were also proven to be effective in MERS patients (De Wilde et al., 2013; Omrani et al., 2014). Severe studies tested and compared the activity of SARS-CoV and MERS-CoV to antiviral drugs. For instance, De Wilde et al. (2013) and De Wit et al. (2016) indicated that SARS-CoV was fifty to one hundred times less sensitive to IFN-α treatment than MERS-CoV (Gu et al., 2005). The therapy of using antibodies targeting the virus was widely recognized during the epidemic of SARS-CoV (Ter Meulen et al., 2004). To date, many antibodies targeting SARS-CoV and MERS-CoV have been identified. However, the difficulties of use worldwide relate to the emergence of possible escape mutants. At present, no approved antiviral therapy is available for SARS or MERS, nor for COVID-19. The drugs available for COVID-19 contain broad-spectrum antiviral drugs such as nucleoside analogues and HIV-protease inhibitors (Lu, 2020). In light of recent studies, broad-spectrum antivirals, including lopinavir, neuraminidase inhibitors, peptides (EK1), and RNA synthesis inhibitors, are considered reasonable for treatment (Rothan & Byrareddy, 2020). To date, a randomized, controlled clinical trial by Ledford et al. found that dexamethasone is first drug shown to save lives and reduce deaths from the COVID-19 (Ledford, 2020). The other drug shown to benefit people who suffered from COVID-19 is the antiviral drug remdesivir. Surprisingly, Remdesivir was testified to have the function of shortening the length of stay (LOS) of people with COVID, but it did not have significant effects on deaths (Oh et al., 2016). In general, both Remdesivir and Dexamethasone are approved in the united states (USA) to treat COVID-19. Besides, many scientists are working diligently to study COVID-19 to gain a better understand of virus-host interactions, in addition to creating novel therapeutics and testing potential vaccines.

## Conclusions and outlook

The novel atypical pneumonia that emerged in Wuhan, Hubei Province has been proven to be caused by SARS-CoV-2, which has homology with SARS-CoV and MERS-CoV to some extent (Liu et al., 2020). The outbreaks of SARS-CoV and MERS-CoV provide us with significant lessons, including valuable experiences and clinical opinions, on how to better fight the SARS-CoV-2 epidemic. Furthermore, the previous treatments can be used as the basis to begin to control the condition. Given the above findings, we present our suggestions. To fully control the situation, we first need to confirm the transmission of SARS-CoV-2 by performing well-designed large-scale case-control studies; then, proper interventions can be taken to control the cause of disease. Second, monitoring signs of viral genome mutations will play a major role in future research. Third, assessing the effects of antiviral drugs by conducting a randomized controlled study (RCT) is also important. Finally, further research on animal vaccines is especially necessary.

## Additional Information and Declarations

### Data Availability

The following information was supplied regarding data availability: This is a review article; there is no raw data.
